# Health and Economic Loss Assessment of PM_2.5_ Pollution during 2015–2017 in Gansu Province, China

**DOI:** 10.3390/ijerph17093253

**Published:** 2020-05-07

**Authors:** Qin Liao, Wangqiang Jin, Yan Tao, Jiansheng Qu, Yong Li, Yibo Niu

**Affiliations:** 1 Key Laboratory of Western China’s Environmental Systems (Ministry of Education), College of Earth and Environmental Sciences, Lanzhou University, Lanzhou 730000, China; liaoqin@llas.ac.cn (Q.L.); liyong17@lzu.edu.cn (Y.L.); 2 Northwest Institute of Eco-Environment and Resources, Chinese Academy of Sciences, Lanzhou 730000, China; jsqu@lzb.ac.cn (J.Q.); niuyb@llas.ac.cn (Y.N.); 3 Key Laboratory for Environmental Pollution Prediction and Control, Gansu Province, College of Earth and Environmental Sciences, Lanzhou University, Lanzhou 730000, China; 4 Institute for Environmental Strategy, Gansu Academy of Eco-environmental Sciences, Lanzhou 730020, China; jinwangqianghky@163.com

**Keywords:** PM_2.5_, health impact, economic loss, underdeveloped region

## Abstract

Many studies have reported that air pollution, especially fine particulate matter (PM_2.5_), has a significant impact on health and causes economic loss. Gansu Province is in the northwest of China, which is a typical economically underdeveloped area. However, few studies have evaluated the economic loss of PM_2.5_ related to health effects in this province. In this study, a log-linear exposure-response function was used to estimate the health impact of PM_2.5_ in 14 cities in Gansu Province from 2015 to 2017, and the amended human capital (AHC) and cost of illness (COI) method were used to evaluate the related economic loss caused by the health impact from PM_2.5_. The results show that the estimated total number of health endpoints attributed to PM_2.5_ pollution were 1,644,870 (95%CI: 978,484–2,215,921), 1,551,447 (95%CI: 917,025–2,099,182) and 1,531,372 (95%CI: 899,769–2,077,772) in Gansu Province from 2015 to 2017, respectively. Correspondingly, the economic losses related to health damage caused by PM_2.5_ pollution were 42,699 (95%CI: 32,380–50,768) million Chinese Yuan (CNY), 43,982 (95%CI: 33,305–52,386) million CNY and 44,261 (95%CI: 33,306–52,954) million CNY, which were equivalent to 6.45% (95%CI: 4.89%–7.67%), 6.28% (95%CI: 4.75%–7.48%), and 5.93% (95%CI: 4.64%–7.10%) of the region Gross Domestic Product (GDP) from 2015 to 2017, respectively. It could be seen that the proportions of health economic loss to GDP were generally high, although the proportion had a slight downward trend. The economic loss from chronic bronchitis and all-cause mortality accounted for more than 94% of the total economic loss. The health impact, economic loss and per capita economic loss in Lanzhou, the provincial capital city of Gansu, were obviously higher than other cities from the same province. The economic loss in Linxia accounted for the highest proportion of GDP. The health impacts in the Hexi region, including the cities of Jiuquan, Jiayuguan, Zhangye, Jinchang and Wuwei, were generally lower, but the economic loss and per capita economic loss were still higher. We also found that urbanization and industrialization were highly correlated with health economic loss caused by PM_2.5_ pollution. In conclusion, the PM_2.5_-related health economic burden in Gansu Province was serious. As an economically underdeveloped region, it was very important to further adopt rigid and effective pollution control policies.

## 1. Introduction

Air pollution is now the world’s largest environmental health risk, and is ranked as the fourth among all factors [1,2]. The vast majority of the world population is exposed to aPM_2.5_ pollution concentration that exceeds the World Health Organization (WHO) Air Quality Guidelines (AQG) levels of 10 μg/m^3^ [2]. The Global Burden of Disease (GBD) study showed that there were about 4.2 million premature deaths related to ambient air pollution across the world [3]. With the rapid industrialization and urbanization, China is also faced with serious air quality issues over the past few decades [4]. In particular, the persistent and heavy haze weather, represented by PM_2.5_, occurs more frequently, is on an unprecedented large scale [5], and has become an important issue affecting China’s environmental quality, public health and social sustainable development [6].The relationship between air pollution and various adverse health outcomes has been studied extensively worldwide [7,8,9,10,11]. Among all kinds of air pollutants, PM_2.5_ is considered to be more toxic and harmful to human health [12], and is most closely related to various health effect endpoints [13]. It can penetrate deep into the lungs and trigger systemic effects, and increase the risk of disease by increasing oxidative stress [14,15]. These adverse effects on human health have caused significant economic and social costs [16,17,18], bringing great pressure to environmental managers and decision makers. Therefore, it is important to evaluate the value of health damage to residents’ exposure to particulate matter. 

A few studies have shown that long-term exposure to PM_2.5_ pollution is associated with premature death from cardiovascular, respiratory and cerebrovascular diseases, as well as the increase in chronic bronchitis, emphysema and asthma [19,20,21,22], which inspires that quantitative assessment of health-related economic loss caused by atmospheric particulate pollution has been carried out in China. These researches focused on the relatively developed regions, such as Beijing, Shanghai, the Beijing–Tianjin–Hebei region, Yangtze River Delta, and the Pearl River Delta region [23,24,25,26,27,28,29,30,31]. Maji et al. [32] and Yang et al. [33] evaluated the health economic loss due to PM_2.5_ of some of the main cities in China, separately. Li et al. [34] estimated that, under four different baseline levels of PM_2.5_, the health economic loss was 4.32–6.32 billion Chinese Yuan in 2015 in Beijing. Wang et al. [35] found that the PM_2.5_-related premature deaths were estimated to be 13,162 (95% CI: 10,761–15,554), and the economic loss was 22.1 (95% CI: 18.1–26.1) billion Chinese Yuan in the Yangtze River Delta (YRD). However, there is still a serious lack of research on the assessment of health economic loss attributed to PM_2.5_-related air pollution in less developed regions, such as Northwest China. Under different socioeconomic conditions, different regions have different sources of PM_2.5_, as well as health effects, resulting in there are also being some differences in health economic loss. Qi et al. [36] estimated the health economic burden, regional distribution and contribution to regional inequality of air pollution in 112 key cities in China from 2003 to 2010, the results of which showed that the more underdeveloped the regional economy, the heavier the health economic burden of pollution would be.

**Figure 1:**
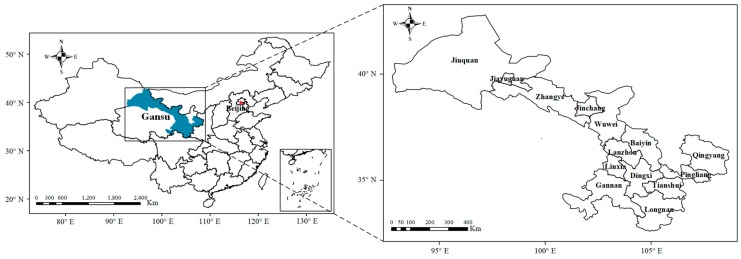
Geographical location of Gansu Province.

Gansu Province is located in the northwest of China (Figure 1), belongs to arid or semi-arid areas, and called the core strategic zone of China’s inland “The Silk Road Economic Belt”. It is often subject to serious particulate pollution that comes from anthropogenic and natural sources. Meanwhile, Gansu Province is a typical underdeveloped region in China, the per capita GDP was 28,497 Chinese Yuan in 2017 [37], ranking the first from the bottom in China. Thus, Gansu Province faces the dual pressure of economic development and environmental protection. To date, research on health effect and economic loss of atmospheric particulate matter (especially PM_2.5_) in the region remains scarce. In this paper, we evaluated the residents’ health risks and the economic loss attributed to PM_2.5_ in Gansu Province from 2015 to 2017, and analyzed the evaluation results of different cities. In order to provide cost-benefit analysis basis or decision-making reference for formulating policies such as air pollution prevention and control to reduce the welfare loss.

## 2. Materials and Methods

### 2.1. Study Areas

Gansu is the most diverse province in natural geographical environment in China, consisting of 14 cities (Figure 1). The types of climate and major industrial structures of economy vary significantly among cities. The climate type of Longnan belongs to “subtropical monsoon climate”, which belongs to the humid region. The cities in the southeast of Gansu Province, including Qingyang, Pingliang, Tianshui, Dingxi and Linxia, have a “temperate monsoon climate” and sub-humid region. The central and northwestern regions of Gansu Province are located in the arid and semi-arid regions. Wuwei, Jinchang, Zhangye, Jiuquan and Jiayuguan have a typical “temperate continental climate”.

The province as a whole can be divided into three traditional economic regions: the Lanzhou metropolitan area, Hedong area and Hexi area (including Jiuquan, Jiayuguan, Zhangye, Jinchang and Wuwei). Lanzhou, located in the central part of Gansu Province, is the capital and the largest city of Gansu Province. It is also a typical heavy industry city in Northwest China, with the highest GDP among all cities in Gansu Province. At the same time, Lanzhou city is located in a long and narrow valley basin, the terrain conditions are relatively closed [38]. Jiayuguan, Jiuquan and Jinchang are also important industrial bases in Gansu Province, with relatively high GDP. Among them, Jiayuguan has the highest per capita GDP in the province. Located in the easternmost part of Gansu, Qingyang is a heavy industry base in the Hedong area, and its GDP is also relatively high, but its per capita GDP is lower than that of the Hexi area. Longnan, Dingxi, Gannan and Linxia are located in the remote loess hilly-gully region or alpine mountainous area, with a relatively weak economic foundation and a low degree of industrialization.

### 2.2. Data Collection

#### 2.2.1. PM_2.5_ Concentrations and Socio-Economic Data

The annual average PM_2.5_ concentrations of 14 cities in Gansu Province during the study period are obtained from the Gansu Provincial Environmental Status Bulletin (2015–2017) published by the Department of Ecology and Environment of Gansu Province (http://sthj.gansu.gov.cn/Department/list.jsp?urltype=tree.TreeTempUrl&wbtreeid=1076). The exposed population is the permanent residents of cities in the study area, which is divided into children (0–14 years of age) and adults (≥15 years of age). The population and GDP data of 14 cities are derived from the Gansu Development Yearbook (2016–2018) [37,39,40].

#### 2.2.2. Exposure–Response Coefficients

The selection of exposure–response coefficients is one of the key links with health risk assessment. Considering health endpoints that have been identified in the existing epidemiological researches, and the availability of the required data, including exposure response coefficient with PM_2.5_, the health endpoints associated with PM_2.5_ exposure are selected in this paper, including all-cause mortality, chronic bronchitis, hospitalization for respiratory diseases, hospitalization for cardiovascular and cerebrovascular diseases, outpatient visits to internal medicine, outpatient visits to pediatrics, and asthma attacks. For the selection of the exposure–response coefficients, due to the level of air pollution in different regions and the sensitivity of different populations to atmospheric particulate matter pollution being different [41], literature containing epidemiological data from Gansu Province is selected in preference, and then the coefficients in other regions of China are applied to analysis when there is a lack of relevant data in the local region, in order to make the exposure–response more reasonable to the actual situation in the study area, and improve the accuracy of this study as much as possible. The selected exposure–response coefficients are shown in Table 1.

#### 2.2.3. Health Information

The mortality data of 14 cities are obtained from the Gansu Development Yearbook (2016–2018). The morbidity of chronic bronchitis is obtained from the China Health and Family Planning Statistical Yearbook (2016) [46]. The hospitalization rates of respiratory diseases and cardio-cerebrovascular diseases are obtained from An Analysis Report of National Health Service Survey in China (2013) [47]. The visiting rate of internal medicine and pediatric, expenses of per capita outpatient and hospitalization, and the loss of working time for hospitalization are based on the average data across Gansu Province, which are obtained from China Health and the Family Planning Statistical Yearbook (2016–2018) [46,48,49]. The loss of working time for outpatient is assumed to be 0.5 days [31,50,51]. The relevant health information of the study area is shown in Table 2.

### 2.3. Estimating Health Effects

The current epidemiological studies on air pollution are mostly based on the relative risk model of Poisson regression [28,53]. Therefore, this paper adopts the exposure–response relationship derived from epidemiological studies to estimate the health loss due to PM_2.5_ exposure. The calculation formula is expressed as:

(1) $$E=E_{0}\times e^{\left[\beta \cdot \left(C-C_{0}\right)\right]}$$

(2) $$N=P\cdot (E-E_{0})=P\cdot E\cdot \left\{1-\frac{1}{e^{\left[\beta \cdot \left(C-C_{0}\right)\right]}}\right\}$$

where *N* represents the number of excess cases or deaths caused by PM_2.5_ pollution, *P* is the exposed population, *E* refers to the incidence of each health endpoint under actual PM_2.5_ concentration (%), *E*_0_ is the incidence of each health endpoint under the baseline PM_2.5_ concentration (%), *β* is the exposure–response coefficient, C refers to the actual PM_2.5_ concentration (μg/m^3^), and *C*_0_ is the baseline PM_2.5_ concentration. This paper selects the World Health Organization Air Quality Guidelines (WHO AQG), i.e., PM_2.5_ is 10 μg/m^3^. 

### 2.4. Economic Loss Evaluation of Health Effects

In this study, the amended human capital (AHC) and cost of illness (COI) approaches are combined to estimate economic loss due to the health damage related to PM_2.5_ pollution.

#### 2.4.1. Amended Human Capital (AHC)

Since the human capital (HC) approach ignores the value of personal health and social happiness, the AHC approach uses per capita GDP to measure the value of a statistical year of life, and is widely used in recent decades. It estimates human capital from the perspective of the entire society, without considering individual differences [24]. AHC is used to calculate the economic loss of PM_2.5_-related all-cause mortality and chronic bronchitis, which is calculated as follows [51,54]:

(3) $$DC_{1}=N\cdot \overset{t}{\underset{i=1}{\sum }}GDP_{pci}^{dv}=N\cdot GDP_{pc0}\cdot \overset{t}{\underset{i=1}{\sum }}\frac{{\left(1+\alpha \right)}^{i}}{{\left(1+\gamma \right)}^{i}}$$

where *DC*_1_ is the economic loss from health impact (all-cause mortality and chronic bronchitis) attributed to PM_2.5_, *N* is the number of all-cause mortality and chronic bronchitis patients caused by PM_2.5_, ${GDP}_{pci}^{dv}$ is the discounted value of per capita GDP in year *I*, *GDP*_*pc*0_ is the per capita GDP in base year, *t* is the average number of life-years lost due to PM_2.5_ pollution, taking 18 years [55], *α* is the growth rate of per capita GDP, and *γ* is the social discount rate, %. According to the literature and the economic development of Gansu Province in recent years, we set *γ* and *α* to 8% and 6%, respectively. For chronic bronchitis, the disability weight of chronic bronchitis (DALY) is about 40% [13,51], that is, 40% of the average human capital is taken as the disability loss.

#### 2.4.2. Cost of Illness (COI)

The COI approach is used to estimate the economic costs of health damage by calculating various disease-related expense, including outpatient visits, hospitalizations and asthma [30,31]. The basic formula is as follows:

(4) $$DC_{2}=\overset{m}{\underset{i=1}{\sum }}(C_{pi}+GDP_{p}\times T_{Ii})\times N_{i}$$

where *DC*_2_ is the economic loss from outpatient visits, hospitalizations and asthma attributed to PM2.5, *C_pi_* represents the direct medical cost per case of health endpoint *i* (outpatient visits, hospitalizations, asthma), *GDP_p_* is the daily per capita GDP of the study city, *T_Ii_* is the working time loss due to health endpoint *I*, *N_i_* is the number of cases of health endpoint *i* caused by PM_2.5_ pollution, *i* is the type of health endpoint, and *m* represents the number of health endpoint. 

## 3. Results

### 3.1. PM_2.5_ Pollution Characteristics

**Figure 2:**
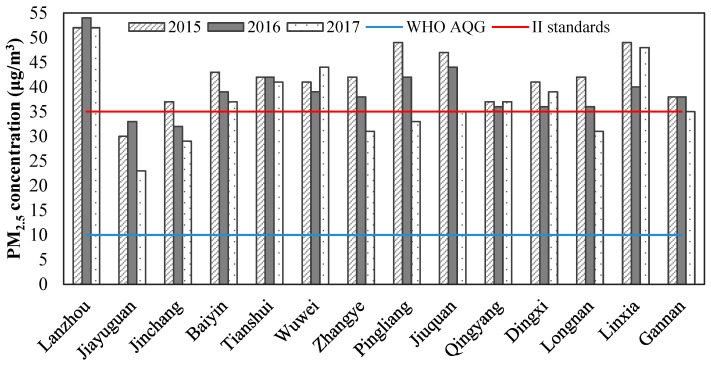
The annual average PM_2.5_ concentration of cities in Gansu from 2015 to 2017.

The annual average concentrations of PM_2.5_ were 42, 39 and 37 μg/m^3^ in Gansu Province from 2015 to 2017, respectively. The annual average concentrations of PM_2.5_ in different cities are shown in Figure 2. It could be seen that the levels of PM_2.5_ in eight cities had gradually decreased from 2015 to 2017. However, the levels of PM_2.5_ in Wuwei, Qingyang, Dingxi and Linxia were lowest in 2016, and they were highest in Lanzhou and Jiayuguan in 2016. In general, the annual average concentrations of PM_2.5_ had shown the largest decline in Pingliang during the study period, followed by Zhangye and Jiuquan.

The annual average concentration of PM_2.5_ in all cities exceeded the value of the WHO AQG (10 μg/m^3^), and most cities exceeded the Secondary Standard Concentration Limit of China’s National Ambient Air Quality Standards (35 μg/m^3^). Even in 2017, only a few cities met the Secondary Standard Concentration Limit of 35 μg/m^3^. Among these cities, Lanzhou suffered the worst serious PM_2.5_ pollution, with the annual average concentration exceeding the national average for the same period from 2015 to 2017, respectively [56]. On the one hand, Lanzhou was located in the valley basin, and the special closed terrain causes static wind and temperature inversion to occur frequently, which was not conducive to the diffusion of pollutants. On the other hand, Lanzhou was one of the largest industrial cities of Northwest China, and, as the capital city of Gansu, the economic development level was higher than that of the other 13 cities, with more coal consumption, car ownership and traffic volume. This could result in larger emissions of pollutants.

### 3.2. Health Effects of PM_2.5_ Pollution

**Figure 3:**
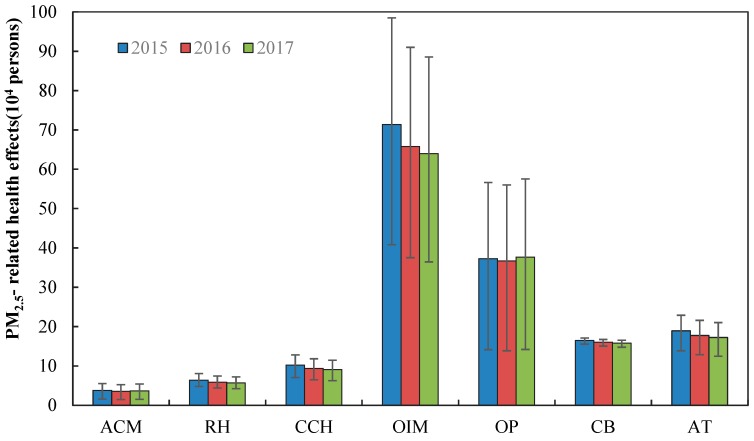
Health effects related to PM_2.5_ in Gansu Province from 2015 to 2017. Note: In Figure 3, ACM, RH, CCH, OIM, OP, CB, AT refer to all-cause mortality, respiratory hospitalization, cardiovascular and cerebrovascular hospitalization, outpatient visits to internal medicine, outpatient visits to pediatrics, chronic bronchitis, asthma attacks, respectively. The same below.

Figure 3 shows the health effects of PM_2.5_ pollution in Gansu Province from 2015 to 2017. The total number of people affected by PM_2.5_ in 2015, 2016 and 2017 was 1,644,870 (95%CI: 978,484–2,215,921), 1,551,447 (95%CI: 917,025–2,099,182) and 1,531,372 (95%CI: 899,769–2,077,772), accounting for 6.4%, 5.9%, and 5.8% of the permanent population at the end of the year, respectively. Therefore, the affected population showed a slightly decreasing trend from 2015 to 2017 all over the province. The proportion of the affected population decreased by 5.68% (95%CI: 5.27%–6.28%) in 2015–2016, larger than the change in 2016–2017.

The sensitivity of health endpoints to PM_2.5_ was different. The number of outpatient visits to internal medicine was the highest, followed by outpatient visits to pediatrics. The number of outpatient visits far exceeded other health effects, and reached 1,068,764 (95%CI: 549,804–1,551,288), 1,025,286 (95%CI: 514,083–1,470,329) and 1,016,486 (95%CI: 506,716–1,460,968) from 2015 to 2017, respectively. Contrastingly, the number of PM_2.5_-related premature mortality was the lowest, with 38,040 (95%CI: 15,958–55,589), 35,662 (95%CI: 14,853–52,455) and 36,722 (95%CI: 15,274–54,082). As opposed to the trend of other health endpoints, the number of outpatient visits to pediatrics increased in 2017.

**Figure 4:**
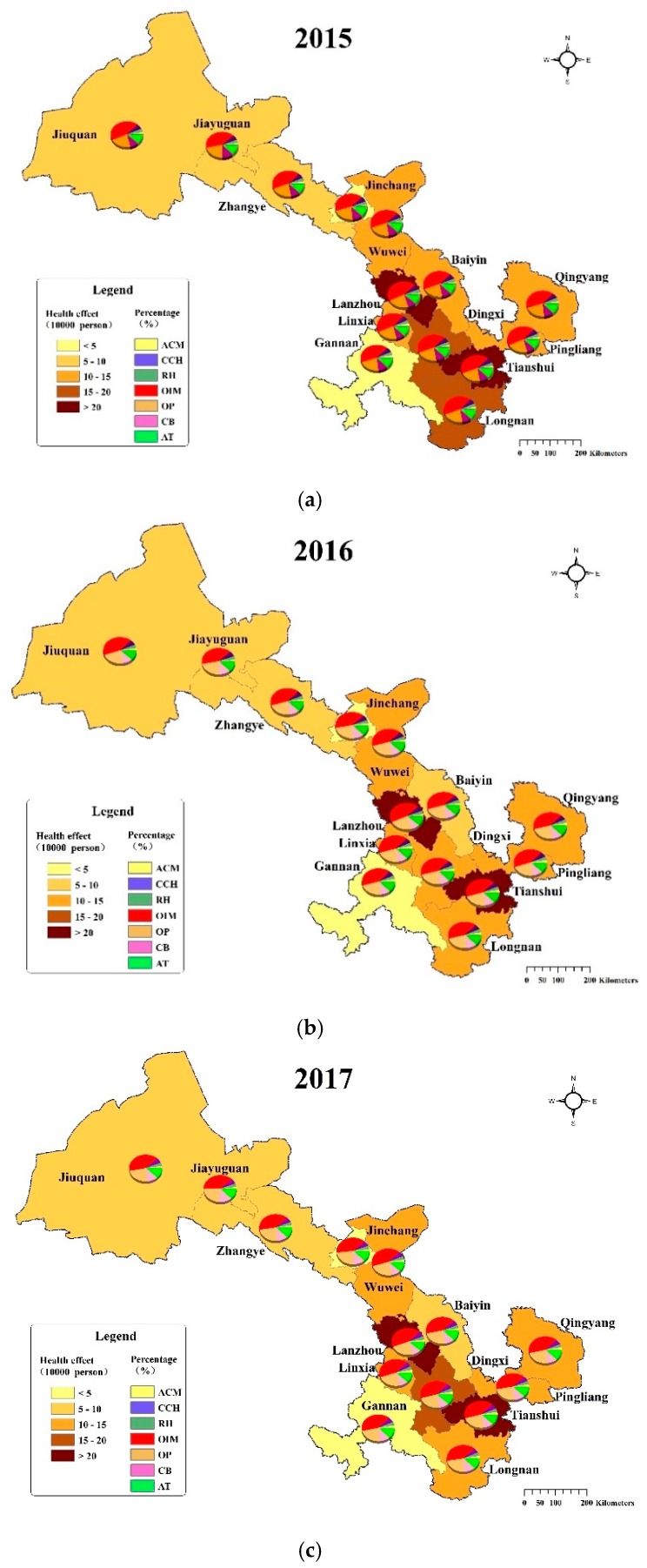
Health effects related to PM_2.5_ in 14 cities in Gansu Province from 2015 to 2017.

We also calculated the health effects of PM_2.5_ in each city in Gansu Province from 2015 to 2017, as shown in Figure 4. Among the 14 cities, Lanzhou and Tianshui were the most affected by PM_2.5_ pollution. The number of people affected by PM_2.5_ from 2015 to 2017 was 276,673 (95%CI: 165,315–370,152), 293,496 (95%CI: 174,455–392,802) and 289,568 (95%CI: 171,066–388,916) in Lanzhou, and 200,635 (95%CI: 119,210–270,877), 200,579 (95%CI: 118,715–271,132) and 204,489 (95%CI: 120,022–277,488) in Tianshui. The affected population in the two cities accounted for 29.02%, 31.85% and 32.26% of the total affected population in Gansu Province from 2015 to 2017, respectively. The affected population in Dingxi, Longnan, Pingliang, Linxia, Qingyang, Wuwei and Baiyin averaged between 100,000 and 200,000. The affected population in Jiuquan and Zhangye was ranging from 50,000 to 80,000. After that, the number of people affected by PM_2.5_ in Gannan and Jinchang was between 20,000 and 40,000. Jiayuguan was the city with the lowest health impact, the affected population was 9827 (95%CI: 5,841–13,395), 11,362 (95%CI: 6,718–15,479) and 7098 (95%CI: 4,167–9,779) from 2015 to 2017, respectively. This was related to the fact that the city had the lowest PM_2.5_ concentration and the smallest population, so the health impact was significantly lower than other cities. 

For different health endpoints, the proportion of health endpoints in overall health effects in 14 cities was similar. Compared with other health endpoints, the proportion of outpatient visits to internal medicine was in the majority, followed by outpatient visits to pediatrics, asthma attacks and chronic bronchitis, while premature mortality, respiratory hospitalization, and cardiovascular and cerebrovascular hospitalization were lower.

According to the annual variation, the number of people affected by PM_2.5_ in nearly half of the cities decreased year by year, including Jinchang, Baiyin, Zhangye, Pingliang, Jiuquan and Longnan. Lanzhou, Jiayuguan and Gannan suffered an increase in health impacts from 2015 to 2016, and then decreased from 2016 to 2017. On the contrary, the health effects were decreased first and then increased in Tianshui, Wuwei, Qingyang, Dingxi and Linxia. 

### 3.3. Economic Loss of PM_2.5_ Pollution

The total estimated economic loss due to PM_2.5_-related health impacts in whole Gansu Province were 42,699 (95%CI: 32,380~50,768) million Chinese Yuan (CNY), 43,982 (95%CI: 33,305~52,386) million CNY and 44,261 (95%CI: 33,306~52,954) million CNY, which accounted for 6.45%, 6.28% and 5.93% of the province’s GDP from 2015 to 2017, respectively (Table 3). It showed that the proportion of GDP for economic loss declined slightly year by year. In each category, the cost caused by chronic bronchitis was the highest, followed by all-cause mortality, and they represented more than 94% of the total economic loss. These findings were generally consistent with the results of previous studies, for instance, Yin et al. (2015) [57], Lv et al. (2016) [13] and Wei et al. (2018) [51].

**Figure 5:**
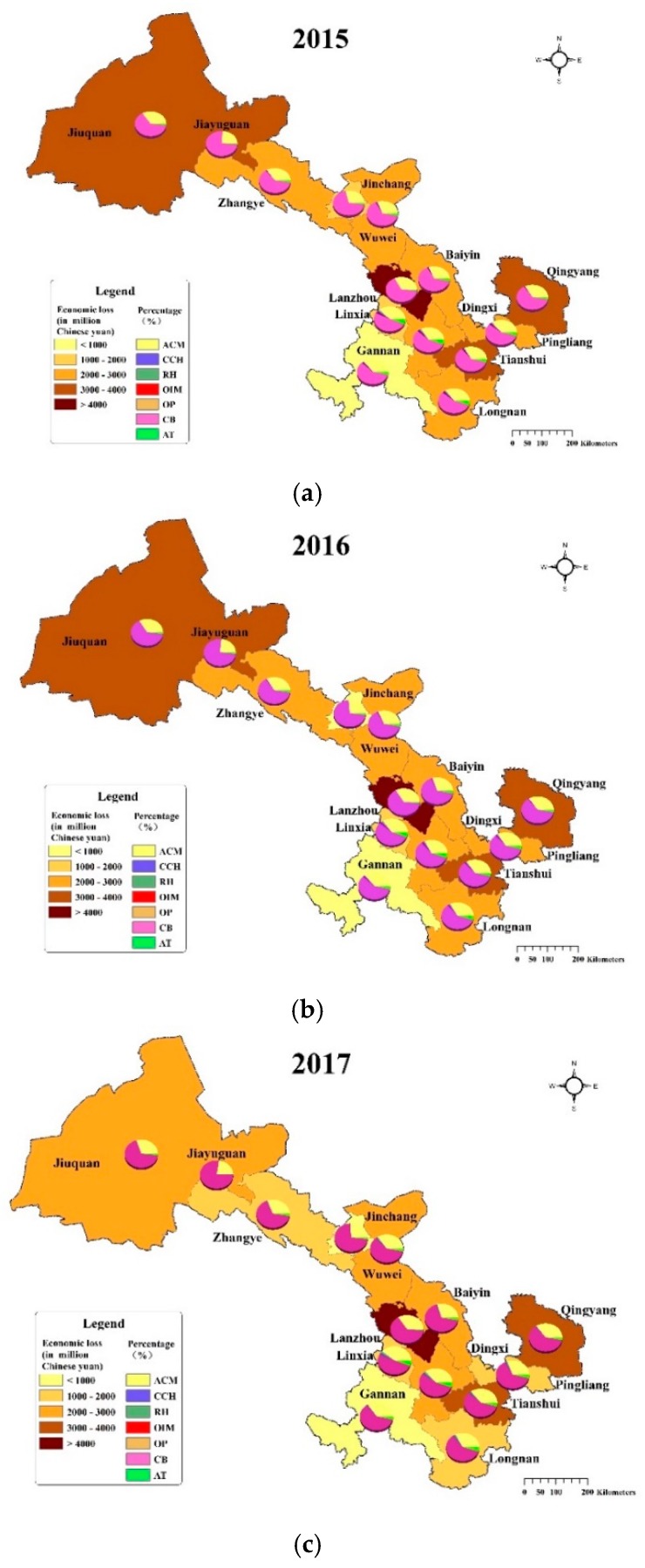
Economic loss of PM_2.5_-related health impacts in 14 cities in Gansu Province from 2015 to 2017.

**Figure 6:**
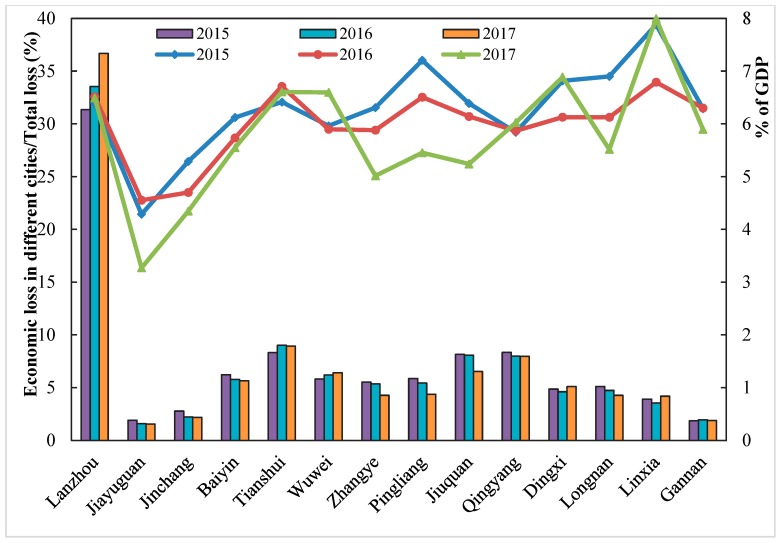
Ratio of economic loss of each city in the total economic loss and the ratio of economic loss to GDP from 2015 to 2017.

Figure 5 displays the distribution of health-related economic loss caused by PM_2.5_ across Gansu Province during 2015–2017. Similar to the health impact, Lanzhou suffered the greatest economic losses, which were 13,387 (95%CI: 10,400–15,635) million CNY, 14,754 (95%CI: 11,439–17,227) and 16,237 (95%CI: 12,506–19,045) from 2015 to 2017, respectively. During the study period, the contribution of economic loss in Lanzhou to the total economic loss in Gansu Province was 31.35%, 33.55% and 36.68%, which was much higher than that of other cities and showed a growing trend (Figure 6). The economic loss was the second highest in Tianshui, Jiuquan and Qingyang, averaging between 3000 and 4000 million CNY. Baiyin, Wuwei, Zhangye, Pingliang, Dingxi and Longnan were the six cities whose economic loss ranged from 2000 to 3000 million CNY in the study period. For Jinchang and Linxia, the economic loss averaged between 1000 and 2000 million CNY. The economic loss of Jiayuguan and Gannan were the lowest, which were less than 1000 million yuan, contributing less than 2% to the total economic loss in Gansu Province.

As can be seen from the pie chart in Figure 5, the proportion of economic loss caused by each health endpoint in the total economic loss also showed a roughly consistent trend in 14 cities. That is, the economic loss caused by chronic bronchitis accounted for the largest proportion, followed by all-cause mortality, and the economic loss of outpatients were the lowest.

From 2015 to 2017, the economic loss in Jiayuguan, Jinchang, Baiyin, Zhangye, Pingliang and Longnan maintained a decreasing trend, while the changes of economic loss showed opposite trends in Lanzhou and Wuwei. In addition, the economic loss in Tianshui, Jiuquan and Gannan increased first and then declined, but Tianshui’s economic loss in 2016 and 2017 was significantly higher than that in 2015, and Jiuquan’s economic loss in 2017 was significantly lower than that in 2015 and 2016. The economic loss in Qingyang, Dingxi and Linxia showed trends of decreasing first and then rising, but the magnitude of the change was slight. 

The proportion of health economic loss to GDP from 2015 to 2017 had been estimated for a parallel comparison in each city. As indicated in Figure 6, on average, Linxia, with more than 7% of GDP loss during 2015–2017, was the highest proportion among all the cities. The ratio in Dingxi, Tianshui, Lanzhou, Pingliang, Longnan, Gannan and Wuwei ranged from 6% to 7%. The economic loss in Jiayuguan was equivalent to around 4% of the GDP, which was the lowest. The ratio of economic loss to GDP was reduced from 2015 to 2017 in nearly half of the cities, for example, Jinchang, Baiyin, Zhangye, Pingliang, Jiuquan, and Longnan. By contrast, the ratios in Lanzhou, Tianshui and Qingyang were relatively stable.

**Figure 7:**
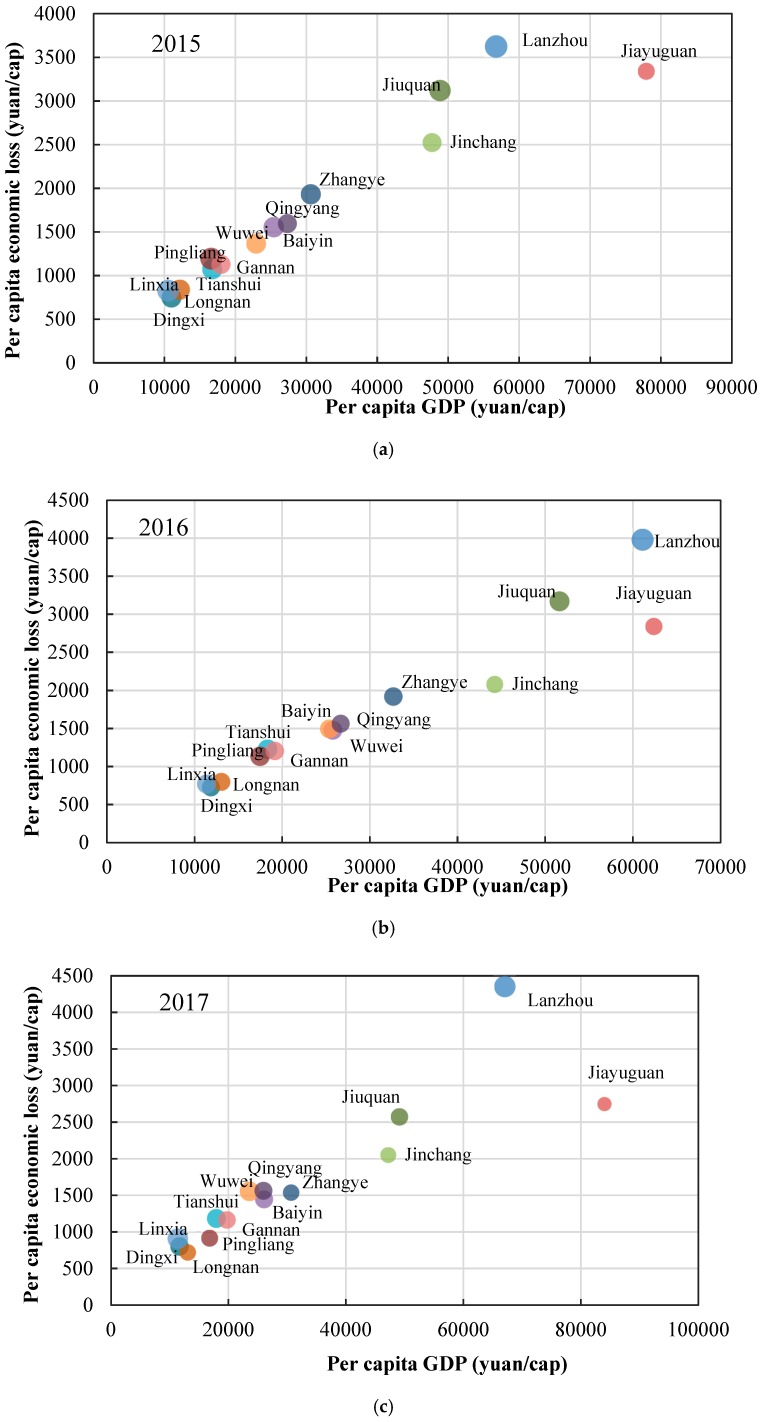
Per capita economic loss in 14 cities in Gansu Province from 2015 to 2017. Note: In Figure 7, the size of the circle represents PM_2.5_ concentration.

In terms of per capita economic loss in 14 cities (Figure 7), it ranged from 721 CNY to 4353 CNY in different cities during 2015–2017. Among them, Lanzhou had the highest per capita economic loss, with more than 3500 CNY per year. The per capita economic loss was more than 2000 CNY in Jiuquan, Jiayuguan and Jinchang. Longnan, Dingxi and Linxia had the lowest per capita economic loss, which was less than 1000 CNY per year. Overall, we could see that the per capita economic loss of cities located in the Hexi area, including Jiuquan, Jiayuguan, Zhangye, Jinchang and Wuwei, was generally significantly higher than other cities. From 2015 to 2017, the per capita economic loss continued to rise in Lanzhou and Wuwei, while that decreased in Jiayuguan, Jinchang, Baiyin, Zhangye, Pingliang, Qingyang and Longnan. Figure 7 also showed that, in addition to the influence of PM_2.5_ pollution, per capita economic loss generally increased with the increase in per capita GDP. 

## 4. Discussions

### 4.1. Correlation Analysis with Social Economic Development

As mentioned in the result part, the health effects of air pollution in different cities had a spatial difference. Previous studies have confirmed that the level of air pollution and its health burden were associated with local socio-economic development [58,59]. In general, the number of people affected by PM_2.5_ in other areas was more than that in the Hexi area of Gansu Province. Larger population density and relatively higher PM_2.5_ concentration were important reasons for the serious health effects in these areas. Therefore, we further discussed the correlation between health effects of PM_2.5_ and socio-economic indicators using correlation analysis (Table 4). It was found that the PM_2.5_ related health effects were significantly positively correlated with the population, GDP, population density and gross industrial production of the region, especially with population and population density. 

In terms of economic loss, Lanzhou, the provincial capital, suffered the highest loss, and much higher than that of other cities. Yang et al. [60] also pointed out that the welfare loss of provincial capital cities was significantly higher than that of other cities in the same province, which was related to the higher consumption level and living quality of capital cities. Correlation analysis showed that the economic loss of PM_2.5_-related health impacts was still significantly positively correlated with the population, GDP, population density, and gross industrial production of the region, especially the extremely significant correlation with GDP and gross industrial production. The GDP and gross industrial production of Lanzhou were indeed significantly higher than that of other 13 cities from the statistical yearbook. 

From the perspective of per capita economic loss, the per capita economic loss was higher in the Hexi area overall, which was contrary to the number of health impacts. This was closely related to the per capita GDP and urbanization rate of in different areas. The per capita GDP and urbanization rate in the Hexi area were both relatively higher, while, in other areas, there were more exposed people and lower GDP, so the per capita GDP was relatively lower.

### 4.2. Policy Implications

The trend of time showed that the air quality had gradually improved in recent years in Gansu Province. The annual average concentration of PM_2.5_ dropped from 42 in 2015 to 37 μg/m^3^ in 2017, and the related health effects also declined year by year. This was largely due to a series of positive air pollution control measures taken in Gansu Province, which mainly involved industrial energy structure, industrial emissions, coal combustion and vehicles. Correspondingly, cities had also introduced relevant policies and measures. In 2015, Lanzhou won the “Today’s Reform Progress Award” jointly granted by the Secretariat of the UN Framework Convention on Climate Change in Paris for its outstanding achievements in air pollution control [61]. In order to further improve the prevention and control of air pollution, the government formulated and issued the Air Pollution Prevention and Control Work Plan in Gansu Province, 2017, and as well as policies on the total emission reduction of major air pollutants and pollution control in the steel and other industrial. In addition, strict monitoring and early warning measures had been implemented. However, although the annual average concentration of PM_2.5_ had decreased year by year, it still exceeded the secondary standard concentration limit of National Ambient Air Quality Standard. The pollution situation was still severe, and more applicable measures should be taken to further combat the problem, especially in cities with serious pollution. 

The absolute value of health economic loss gradually increased in Gansu Province from 2015 to 2017, but the proportion of economic burden (as a proportion of GDP) showed a downward trend. Meanwhile, the expenditure on pollution prevention and control increased from 0.21% in 2015 to 0.27% in 2017 (the total expenditure on energy conservation and environmental protection was averaged about 1.4% per year during 2015–2017 period) in Gansu Province [37,39,40]. Hence, it was also important for the local government to increase investment in environmental protection from the aspects of both protecting public health and sustainable economic development. 

### 4.3. Uncertainty Analysis

In the selection of health endpoints, the study mainly chose health endpoints that could be quantitatively evaluated, such as all-cause mortality, chronic bronchitis, hospitalization for respiratory diseases, hospitalization for cardiovascular and cerebrovascular diseases, outpatient visits to internal medicine, outpatient visits to pediatrics, and asthma attacks. However, as for the lack of relevant epidemiological studies and available basic data, other health endpoints were ignored, including lung function changes and mental illnesses that had been shown to be associated with PM_2.5_ [62,63,64]. Thus, it was not comprehensive enough to measure all health endpoints caused by PM_2.5_, which probably underestimated the health impact and economic loss of PM_2.5_ pollution.

The exposure–response coefficient of PM_2.5_ depended on the results of epidemiological and toxicological studies. At present, many other factors in the process of establishing the exposure– response relationship in relevant studies were still unclear and uncertain, and further studies were needed. In addition, the exposure–response coefficient varied by different regions and cities. The higher the exposure–response coefficient, the higher the health impact would be. For example, in this study, the exposure–response coefficient of all-cause mortality was selected from the local study results in Lanzhou. It was higher than that of other regions in China, and the estimated number of all-cause mortality was relatively high. Therefore, the exposure–response coefficient of the local or similar region should be prioritized to avoid errors caused by regional differences, so as to improve the accuracy of the study as far as possible.

There was no scientific basis for setting a specific threshold currently. In the assessment of the health risks of air pollution, the selection of a threshold usually included no threshold (or zero threshold), natural background values, the lowest concentration values observed in the epidemiological literature, as well as the air quality standards released by government or AQG released by WHO [65]. The lower safe threshold of air pollution was used in our study, that is, the AQG was used as the threshold concentration, because it was based on a large amount of scientific evidence currently associated with air pollution and its health effects. Most studies have also used this threshold. It was clear that the results of health economic loss were also sensitive to the threshold of PM_2.5_ concentration. Taking the city of Lanzhou as an example, according to our calculation, the economic loss accounted for 6.39%, 6.52% and 6.49% of GDP under the reference concentration of 10 μg/m^3^ from 2015 to 2017, respectively, and 3.96%, 4.25% and 4.01% under the reference concentration of 35 μg/m^3^ (national secondary standard), respectively. Therefore, reducing PM_2.5_ concentration to meet stringent air quality standards could reduce health damage and avoid a large quantity of economic loss [66].

Furthermore, we did not consider the spatial differences of PM_2.5_ concentrations and population distribution within the city when calculating the health effects of PM_2.5_ pollution in each city. Another point was that, although the morbidity, per capita hospitalization and outpatient costs of different diseases varied in different cities, the average health information of Gansu Province was used when the information for disease incidence rate in a city was not available, due to the data availability. In conclusion, the selection of health endpoints, exposure–response coefficient, threshold concentration of PM_2.5_ and baseline level all influenced the results of the study.

## 5. Conclusions

We conducted a spatio-temporal assessment of the economic loss caused by PM_2.5_-related health effects in cities of Gansu Province, combining epidemiological methods with economic methods.

The number of health damage caused by PM_2.5_ pollution was 1,644,870 (95%CI: 978,484–2,215,921), 1,551,447 (95%CI: 917,025–2,099,182) and 1,531,372 (95%CI: 899,769–2,077,772) in Gansu Province from 2015 to 2017, respectively, and it could be drawn that there were downward trends year by year. Among the different health endpoints, PM_2.5_ pollution had the strongest impact on outpatient visits (internal medicine and pediatrics). For a single city, Lanzhou with the highest PM_2.5_ concentration and most exposed population suffered the highest health impacts, while the health impact in Jiayuguan was the lowest. The health impacts in Hexi area were generally lower than that in other areas.

The economic value of health loss caused by PM_2.5_ were 42,699 (95%CI: 32,380–50,768) million CNY, 43,982 (95%CI: 33,305–52,386) million CNY and 44,261 (95%CI: 33,306–52,954) million CNY in Gansu from 2015 to 2017, respectively. On the whole, although the proportion of economic loss in the total GDP declined slightly, it remained high, accounting for 6.45% (95%CI: 4.89%–7.67%), 6.28% (95%CI: 4.75%–7.48%) and 5.93% (95%CI: 4.64%–7.10%) of GDP for that year, respectively. Chronic bronchitis and all-cause mortality were the main sources of total economic loss, with a combined contribution rate of more than 94%. Among the 14 cities, Lanzhou had the highest economic loss, equivalent to about one third of the total health economic loss of Gansu Province, and the highest per capita economic loss. Linxia suffered the greatest health economic loss in terms of the proportion of local GDP.

Despite the uncertainties, according to the quantitative evaluation results, the health-related economic loss caused by PM_2.5_ pollution accounted for a high proportion in the GDP, highlighting the severity of the health effects caused by PM_2.5_ pollution in Gansu Province. It brought greater pressure on the sustainable development of local economy and society, due to the low level of economic development. Hence, efforts should be made to strengthen the control of air pollution in the region, in order to minimize the health hazards and economic loss caused by air pollution.

## Figures and Tables

**Table 1:** Exposure-response coefficients of fine particulate matter (PM_2.5_) related health effects.

Health Outcome	Exposure–Response Coefficient *β* (95%CI)
All-cause mortality	0.0083 (0.0032, 0.0131) [42]
Respiratory hospitalization	0.00604 (0.00441, 0.00789) [43]
Cardiovascular and cerebrovascular hospitalization	0.00697 (0.00464, 0.00906) [43]
Outpatient visits to internal medicine	0.0049 (0.0027, 0.007) [44]
Outpatient visits to pediatrics	0.0056 (0.002, 0.009) [44]
Chronic bronchitis (0–14)	0.0486 (0.0401, 0.0570) [13]
Chronic bronchitis (≥15)	0.0686 (0.0574, 0.0797) [13]
Asthma attacks (0–14)	0.0208 (0.0118, 0.0300) [45]
Asthma attacks (≥15)	0.021 (0.0145, 0.0274) [45]

**Table 2:** The health information in Gansu.

Year		2015	2016	2017
Mortality (‰)		Mortality Rate per City [37,39,40]		
Morbidity (‰)	Chronic bronchitis (0–14)	7.2 [46]	7.2	7.2
	Chronic bronchitis (≥15)	7.2	7.2	7.2
	Asthma attacks (0–14)	23.9 [52]	23.9	23.9
	Asthma attacks (≥15)	12.4 [51]	12.4	12.4
Visiting Rate (‰)	Outpatient visits to internal medicine	179.8 [46]	180.6 [48]	181.3 [49]
	Outpatient visits to pediatrics	83.1 [46]	89.1 [48]	94.4 [49]
Hospitalization Rate (‰)	Respiratory hospitalization	13.3 [47]	13.3	13.3
	Cardio-cerebrovascular hospitalization	18.7 [47]	18.7	18.7
Medical Costs Per Case (CNY/case)	outpatient costs	169.7 [46]	177.4 [48]	190.9 [49]
	hospitalization costs	5447.1 [46]	5587.5 [48]	5769.8 [49]
Loss of Working Time (day)	average outpatient time	0.5	0.5	0.5
	average hospitalization time	9.7 [46]	9.1 [48]	8.8 [49]

**Table 3:** Economic loss of PM_2.5_-related health impacts in Gansu Province from 2015 to 2017.

Health Outcome	Economic Loss of Health Effects (in Million CNY)		
	2015	2016	2017
ACM	14,213.83	14,601.64	14,856.45
	(5980.11, 20,719.46)	(6125.30, 21,343.40)	(6214.83, 21,771.20)
RH	393.28	371.14	368.74
	(295.69, 499.14)	(278.26, 471.55)	(276.46, 469.18)
CCH	628.27	593.52	589.92
	(435.73, 790.49)	(410.15, 747.76)	(407.54, 744.37)
OIM	147.31	143.38	148.15
	(84.51, 203.76)	(81.95, 198.48)	(84.68, 205.49)
OP	76.90	79.96	87.22
	(29.28, 117.14)	(30.29, 122.06)	(33.02, 133.48)
CB	26075.91	27,073.51	27,095.92
	(24,701.58, 27,029.60)	(25,565.71, 28,141.09)	(25,480.61, 28,267.91)
AT	1163.96	1118.51	1115.04
	(853.48, 1408.56)	(813.56, 1361.67)	(809.22, 1362.10)
Total	42,699.46	43,981.65	44,261.44
	(32,380.38, 50,768.15)	(33,305.22, 52,386.00)	(33,306.36, 52,953.74)
Total/GDP	6.45%	6.28%	5.93%
	(4.89%, 7.67%)	(4.75%, 7.48%)	(4.47%, 7.10%)

Note: ACM, RH, CCH, OIM, OP, CB, AT refer to all-cause mortality, respiratory hospitalization, cardiovascular and cerebrovascular hospitalization, outpatient visits to internal medicine, outpatient visits to pediatrics, chronic bronchitis, asthma attacks, respectively.

**Table 4:** Correlation between PM_2.5_ related health impact, economic loss and socioeconomic indicators.

Category	Year	Population	GDP	Per Capita GDP	Urbanization Rate	Population Density	Gross Industrial Production
Health Impact	2015	0.977 **	0.706 **	−0.312	−0.184	0.813 **	0.536 *
	2016	0.959 **	0.802 **	−0.082	−0.088	0.791 **	0.703 *
	2017	0.953 **	0.747 **	−0.164	−0.129	0.811 **	0.650 *
Economic Loss	2015	0.645 *	0.998 **	0.290	0.366	0.548 *	0.915 **
	2016	0.650 *	0.999 **	0.438	0.361	0.552 *	0.945 **
	2017	0.648 *	0.997 **	0.361	0.361	0.574 *	0.948 **
Per Capita Economic Loss	2015	−0.263	0.525	0.955 **	0.927 **	−0.092	0.601 *
	2016	−0.103	0.655 *	0.958 **	0.855 **	−0.009	0.675 **
	2017	0.001	0.764 **	0.879 **	0.854 **	0.130	0.811 **

* Correlation is significant at the 0.05 level (*p* < 0.05); ** Correlation is significant at the 0.01 level (*p* < 0.01).
